# Epidermal growth factor mediates spermatogonial proliferation in newt testis

**DOI:** 10.1186/1477-7827-6-7

**Published:** 2008-02-06

**Authors:** Keisuke Abé, Ko Eto, Shin-ichi Abé

**Affiliations:** 1 Department of Biological Sciences, Graduate School of Science and Technology, Kumamoto University, 2-39-1 Kurokami, Kumamoto 860-8555, Japan

## Abstract

The complex processes of spermatogenesis are regulated by various factors. The aim of the current study is to determine the effect of epidermal growth factor (EGF) on spermatogonial proliferation and clarify the mechanism causing the proliferation in newt testis. In the organ culture, EGF stimulated spermatogonial proliferation, but not their differentiation into spermatocytes. cDNA cloning identified 3 members of the EGF receptors, ErbB1, ErbB2, and ErbB4, in the testis. RT-PCR showed that all the receptors cloned were expressed in both Sertoli and germ cells at the spermatogonial stage. In the organ cultures with inhibitors for the EGF receptors, mitogen-activated protein kinase (MAPK), and phosphoinositide 3-kinase (PI3K), the EGF-induced spermatogonial proliferation was suppressed. Furthermore, when the organ culture was exposed to EGF, the expressions of stem cell factor (SCF), immunoglobulin-like domain containing neuregulin1 (Ig-NRG1), and ErbB4 mRNA were increased. These results suggested that, since the spermatogonia are sequestered within cysts by the blood-testis barrier consisted of Sertoli cells, EGF possibly mediates spermatogonial proliferation in an endocrine manner through the receptors including ErbB1, ErbB2, and ErbB4 expressed on Sertoli cells via activation of MAPK cascade or/and PI3K cascade by elevating the expressions of SCF, Ig-NRG1, and ErbB4.

## Background

Spermatogenesis is a complex process consisting of sequential and highly organized steps of germ cell proliferation and differentiation, resulting in the generation of functional spermatozoa, in the testis [1]. A wide range of hormones and growth factors regulate these processes in an endocrine manner mainly through Sertoli cells, a kind of somatic cells interacting directly with germ cells in the testis, eventually [2]. Sertoli cells have essential roles in the spermatogenic function of the testis: they produce and secrete local factors to germ cells, and represent the only cellular component of the blood-testis barrier [3]. In newt testis, the germ cells are in close contact with Sertoli cells in a cyst, the smallest unit of the testis, and the testis consists of lobules in successive zones arranged along a cephalo-caudal axis, in which spermatogenesis proceeds synchronously [4,5]. Primary spermatogonia proliferate through 7 mitotic divisions (the spermatogenic stage mentioned here is largely classified into early spermatogonial stages (1st – 4th generation) and late spermatogonial stages (5 – 7th generation)), and then in the 8th generation differentiate into primary spermatocytes and initiate meiosis. Recently, we have demonstrated with the organ culture that the functional blood-testis barrier with size selectivity, allowing small molecules (~500 Da) to get into cysts, but not larger ones (> 1.9 kDa), consists of Sertoli cells in the testis [6].

Of all the hormones involved in spermatogenesis, follicle-stimulating hormone (FSH) plays a determinant role in stimulating spermatogonial proliferation and differentiation in addition to increasing the survival of germ cells [7]. The FSH effects on germ cells are mediated through the specific receptor expressed restrictedly in Sertoli cells [8], by which paracrine factors are necessary to be produced and secreted locally to act directly on germ cells within the testis. In mammalian testis, it has been first found that the receptor tyrosine kinase c-kit and its ligand stem cell factor (SCF) are expressed in germ cells and in Sertoli cells, respectively [9,10], and that SCF is upregulated upon FSH stimulation [11]. The interaction of c-kit and SCF is important for the maintenance and/or mitosis of differentiating type A spermatogonia [12], defining SCF as a paracrine factor in the regulation of spermatogenesis.

In newt testis, we have shown so far that (1) FSH stimulates spermatogonial proliferation and their differentiation into primary spermatocytes in the organ culture [13,14] and in the reaggregated culture of spermatogonia and Sertoli cells in a synthetic medium but not in culture of spermatogonia alone [15], (2) FSH receptor is expressed in Sertoli cells [16], and (3) the intracellular level of cyclic AMP, a probable second messenger in FSH signaling, is elevated in cultured Sertoli cells [17]. These findings strengthen the idea that FSH activates Sertoli cells that consequently produce some paracrine factors necessary for triggering spermatogonial proliferation and differentiation. In fact, we have identified some paracrine factors including SCF [18], insulin-like growth factor-1 [19], and neuregulin1 (Oral et al., submitted) that are upregulated by FSH and implicated in the regulation of spermatogonial proliferation during newt spermatogenesis.

Epidermal growth factor (EGF), a polypeptide comprising 53 amino acid residues, was originally isolated and purified from the submandibular glands of adult male mice [20]. High levels of EGF are detected in the circulation [21]. Mouse EGF is exclusively produced in and secreted from the submandibular gland. However, it was reported to be produced in many other tissues including testis, where immunoreactivity for the mature type of EGF is detected in Sertoli cells, pachytene spermatocytes, and round spermatids, while that for the precursor type of EGF was limited to pachytene spermatocytes and round spermatids [22]. In porcine testis, it is produced in Leydig cells [23] and plays an important role in their physiology and pathophysiology [24]. In other mammalian species, EGF was localized in both germ cells and somatic cells stage-dependently [25,26]. After initiation of stage synchrony of spermatogenesis, increased concentrations of EGF were observed in rat testis between stages IX-II that correlated well with mitotic division of type A spermatogonia [27]. The EGF levels in the testis were shown to correlate with testicular mass, number of spermatozoa, and production of haploid cells [28]. Therefore, it seems that proper EGF expression is necessary for the completion of spermatogenesis [29].

EGF is well known to stimulate cell proliferation and differentiation in a variety of tissues. In adult male mice, submandibular gland ablation caused a marked decrease in circulating EGF levels and male fertility, without diminishing testosterone and FSH, and correlated with over a 50% decrease of epididymal spermatozoa, which was reversed by daily administration of EGF [30]. Similar results were also reported in rat [31]. In addition, the average concentration of EGF in blood plasma is significantly lower in infertile males [32]. In mammalian testes, EGF also promotes spermatogonial proliferation and modulates steroidogenesis, spermiogenesis, proliferation of Leydig cells, Sertoli cell activity in an autocrine and a paracrine manner [23,33]. In the organ culture of cryptorchid testis from adult mice, EGF induces differentiation of type A spermatogonia to type B spermatogonia and primary spermatocytes, but inhibits the mitotic and differentiating activity stimulated by FSH [34]. EGF stimulates DNA synthesis of spermatogonia in the culture of microdissected stage I segments of rat testis seminiferous tubules [35]. Thus, EGF is probably implicated in the regulation of mammalian spermatogenesis. However, little is known about the mechanism of action of EGF on newt testis, that is, whether its effects are caused by direct action on spermatogonia or by indirect action via somatic cells, and its functions in newt spermatogenesis, that is, whether it can stimulate spermatogonial proliferation and differentiation.

EGF and other EGF-like peptide family members act through the EGF receptors, ErbB family of tyrosine kinase receptors, which consist of four members, ErbB1 (also known as EGFR) [36], ErbB2 [37], ErbB3 [38,39], and ErbB4 [40]. These receptors have been reported to be involved in the regulation of proliferation and differentiation in many tissues [41]. The EGF receptors are known to form homodimers or heterodimers, and signal mainly through mitogen-activated protein kinase (MAPK) or/and phosphoinositide 3-kinase (PI3K) signaling pathway [42,43]. In mammalian testis, all the members of the EGF receptors are identified in various cell types such as germ cells and Sertoli cells and at all spermatogenic stages, suggesting that they are potentially responsive to EGF during spermatogenesis and postnatal testis development, whereas their differential expressions are found in Leydig, Sertoli, and peritubular cells [35].

In the present study, in order to determine the role of EGF in spermatogonial proliferation and differentiation during newt spermatogenesis, we examined whether the addition of recombinant human (rh) EGF to the culture of testicular fragments stimulated 5-bromo-2-deoxyuridine (BrdU) incorporation and appearance of spermatocytes. Next we isolated partial cDNA clones for the EGF receptors, ErbB1, ErbB2, and ErbB4, from newt testis, and examined their mRNA expressions by RT-PCR. Furthermore, we examined the effects of some inhibitors for ErbB, PI3K, and MAPK on EGF-induced spermatogonial proliferation to clarify the receptors and signaling pathways operated by EGF. Finally, in order to explore the molecular mechanism by which spermatogonial proliferation was stimulated in response to EGF, we tested the effect of rhEGF on the gene expressions of SCF, c-kit, neuregulin1, and ErbB family members, which are implicated in spermatogonial proliferation, in the organ culture.

## Methods

### Animals and inhibitors

Animal experiments have been carried out under the control of the Guideline to Animal Experiment in Kumamoto University. Adult male newts, *Cynops pyrrhogaster*, were purchased from Hamamatsu Seibutsu Kyozai Ltd. (Hamamatsu, Japan) and kept at 8°C. Prior to be used for all the experiments, newts were transferred to 22°C and fed frozen *Tubifex *for 1 week. PD98059, a MAPK inhibitor, and AG879, an ErbB2 inhibitor, were purchased from Calbiochem, AG1478, an EGFR inhibitor, and Wortmannin and LY294002, PI3K inhibitors, from Sigma, and PD153035, a pan ErbB inhibitor, from Biaffin (Kassel, Germany).

### Organ culture of testicular fragments, histology, and BrdU incorporation assay

The immature portions containing late spermatogonial stage (5 – 7th generation) were removed from the whole testes and cut transversely and longitudinally into several pieces (1 – 2 mm in thickness). They were cultured on floaters of nucleopore filters (Whatman, Cyclopore™ Track Etched Membrane) in 2 ml of Leibovitz's (L)-15 medium (70% tonicity for mammals, Gibco) supplemented with 10 mM HEPES/NaOH, pH 7.4, at 18°C in a humidified incubator in the absence or presence of either porcine FSH at 200 ng/ml (National Hormone & Peptide Program, West Carson, CA) or rhEGF (Sigma) at various doses as indicated. The pieces were fixed in Bouin's fixative, embedded in paraffin wax, and serially sectioned at 5 μm. The sections were stained with hematoxylin and eosin. After the fragments were cultured for 2 weeks, differentiation of spermatogonia into primary spermatocytes was evaluated in the sections by histological observation [5].

Proliferation of spermatogonia was assayed in the sections by immunohistochemical detection of BrdU incorporated into replicating DNA in the cells. After cultured for 1 week, the fragments were labeled for 6 hrs by addition of BrdU and processed for immunohistochemistry according to manufacturer's instructions (GE Healthcare). For quantification of spermatogonial proliferation, a minimum of 300 – 500 cysts from at least 3 independent sections was examined for BrdU incorporation because all the spermatogonia in a given cyst incorporate BrdU synchronously into DNA during replication [44]. The frequency of proliferation was expressed as a percentage (means ± SEM) of BrdU positive cysts among live ones in 3 sections obtained from 3 independent experiments.

### Spermatogenic staging, and fractionation of germ cells and somatic cells

The immature portions of testes containing early spermatogonial, late spermatogonial, or primary spermatocyte stage exclusively was cut transversely into several pieces (1 – 2 mm in thickness) and then each piece was cut longitudinally (cephalo-caudally) into halves as described previously [45]. A half of each piece was processed for histology, and the counterpart was used for RNA extraction followed by reverse transcription and polymerase chain reaction (RT-PCR).

The testicular portions containing spermatogonial stage were cut into small fragments and dissociated by incubation in 0.1% collagenase (type N-2, Nitta Zeratin Co. Japan) for 3 hrs at room temperature and 1.5 kU/ml DNase I (type IV, Sigma) for the last 5 min in L-15 medium followed by pipetting. The cell suspension was washed in L-15 medium and the undissociated cell clumps were removed by filtration through nylon gauze (50 μm). To remove dead cells, reticulocytes, and mature sperm, the dissociated cells in L-15 medium (5 ml) were put on 15% Nycodentz (Sigma) in L-15 medium (5 ml) and centrifuged at 1,500 *g *for 10 min (HIMAC CT5DL, HITACHI). The layer formed at the interface between L-15 medium and Nycodentz solution containing live spermatogonia and somatic cells was recovered and washed in L-15 medium. The spermatogonia and somatic cells recovered were then separated by culturing on gelatin-coated dishes overnight. The supernatants containing spermatogonia in the dishes were gently transferred into new gelatin-coated dishes and then cultured overnight to remove contaminated Sertoli cells. Next day, the supernatant were gently recovered and centrifuged at 1,000 rpm for 10 min, resulting in collecting spermatogonia. On the other hand, Sertoli cells attached on the dishes were kept culturing at 25°C in L-15 medium supplemented with 10% fetal bovine serum for 10 days and 1 month to remove contaminated spermatogonia thoroughly. Spermatogonial and Sertoli cell fraction, each of which purity was higher than 90%, were processed for RT-PCR.

### cDNA cloning and RT-PCR

The cDNA clones encoding ErbB1, ErbB2, and ErbB4 in newt testis were isolated by RT-PCR. Total RNA was extracted from the immature portions containing spermatogonial stage of the testes, which had been homogenized in ISOGEN (Nippon Gene) using a Dounce homogenizer, and cDNA was reverse transcribed with oligo-d(T) primers by a reverse transcriptase Superscript III (Invitrogen), as described previously [46]. PCR was performed by ExTaq polymerase (Takara) or Go Taq polymerase (TOYOBO) in the condition for 45 cycles at 95°C for 0.5 min, 55°C for 0.5 min, and 72°C for 1 min using reverse transcribed cDNA as a template with a sense and an antisense primer that were designed on the basis of the nucleotide sequences of ErbB1, ErbB2, and ErbB4 from human, mouse, and rat in NCBI database, respectively, as follows: ErbB1, 5'-CCA CGA GCA CAA GGA TAA CA-3' and 5'-CAC TCC AGA GCC ATC CAT TT-3'; ErbB2, 5'-ATG TCC GGG AAC ACA AAG AC-3' and 5'-GAT TCC AAT GCC ATC CAC TT-3; ErbB4, 5'-CTG CAC GAG ACT AGT GAG AC-3' and 5'-TGT GCG CAG GAA CAG AGA AC-3'. Each of the amplified DNA fragments was inserted into pT7Blue vector (Novagen). The nucleotide sequence was completely determined using an Applied Biosystems model 310 automated DNA sequencer.

Expressions of mRNA for SCF, c-kit, 3 members of the EGF receptor, ErbB1, ErbB2, and ErbB4, and 2 isoforms of neuregulin1, Ig-NRG1 and CRD-NRG1, at the early spermatogonial, late spermatogonial, and spermatocyte stage, and in the spermatogonia and Sertoli cells were analyzed by RT-PCR. Total RNA was extracted from the testicular portions containing the respective spermatogenic stage and the testicular cells fractionated, and cDNA was reverse transcribed with random hexamers. PCR was performed in the condition for cycle numbers as indicated at 95°C for 30 sec, 53°C for ErbB1, ErbB2, and ErbB4, and 55°C for SCF, c-kit, EF-1α, Ig-NRG1, and CRD-NRG1 for 30 – 60 sec, and 72°C for 60 sec with a sense and an antisense primer specific for each of cDNA clones isolated from newt as follows: ErbB1, 5'-GCC AAC AAG GAA ATT CTG GA-3' and 5'-CCC TTT GCA ATC TGA ACA CA-3'; ErbB2, 5'-TCA CAG GAC CTG CTC AAC TG-3' and 5'-GTC GAG CGA GTC CAA AGT CT-3'; ErbB4, 5'-TTG GTG TGT CCC AGA TAG CC-3' and 5'-GCG CTG TAC TCC TTC TCG TC-3'; SCF, 5'-GTG TAA CTT TCG GAA ATC CAT GCG G-3' and 5'-ACT TCT TCG GGA CAA ACT GAC CCT C-3'; c-kit, 5'-CTC ACT CGT GGA CGC ATT ACA AAG A-3' and 5'-TTC CAT ATG ACC AGA CAT CGC TCT C-3'; Ig-NRG1, 5'-GCT GGT GCT GAA GTG TCA AG-3' and 5'-GTG CAT CTT GCT CCA GTG AA-3'; CRD-NRG1, 5'-CCT TTA TAC TGA CAC AGC TCC-3' and 5'-GGT GTC ACC CCT TTT GGT TG-3'; EF-1α, 5'-AGC CCT AGA CTC AAT CAT CC-3' and 5'-ATC CAA CAC AGG AGC GTA TC-3'.

### Statistics

Data were obtained as the means ± SEM from at least 3 independent experiments.

For statistical comparison, Student't *t *test was used. Probability (P) values less than 0.05 were considered to be statistically significant.

## Results

### Effect of EGF on the proliferation and differentiation of spermatogonia in the testis

**Figure 1:**
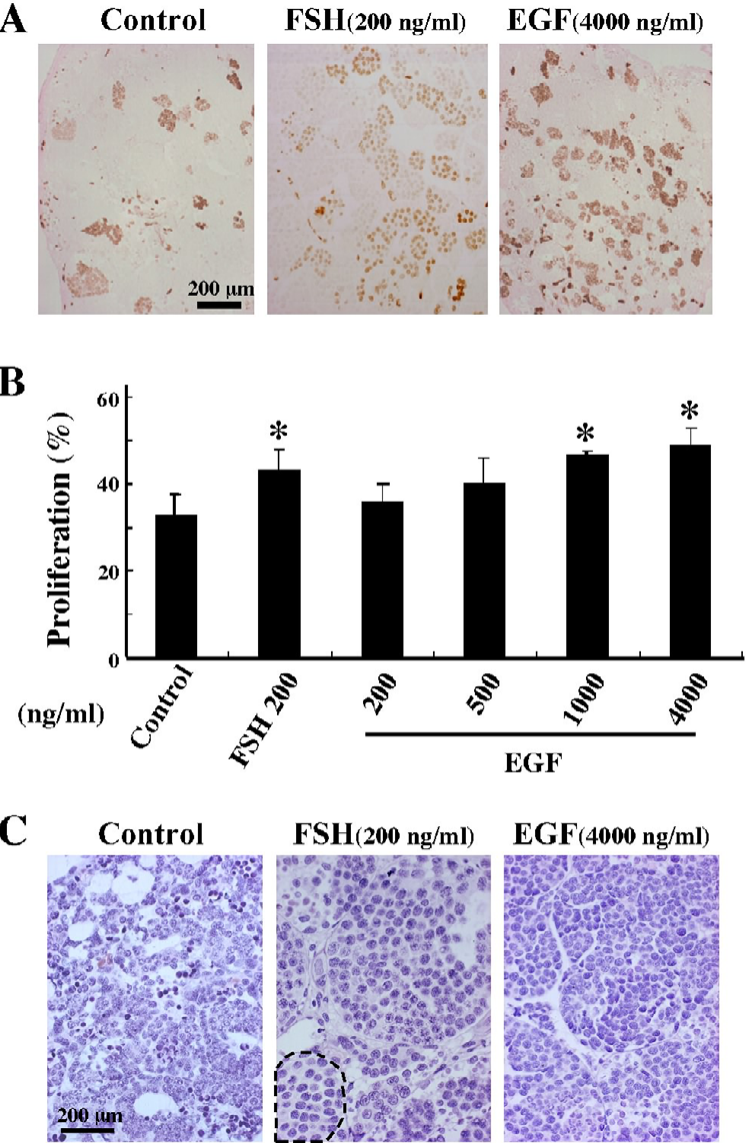
Effect of EGF on spermatogonial proliferation and their differentiation to primary spermatocytes. (A and B) Stimulatory effect of EGF on spermatogonial proliferation. Testicular fragments containing speramatogonial stage were cultured for 1 week in the absence or presence of either FSH or various doses of EGF indicated. (A) Immunohistochemistry for BrdU incorporation in the sections of the fragments treated without (Control) or with either FSH or EGF. (B) Spermatogonial proliferation was determined by counting BrdU positive cysts among live ones in at least 3 sections. *, P < 0.05. (C) No effect of EGF on spermatogonial differentiation. Testicular fragments containing spermatogonial stage were cultured for 2 weeks in the absence (Control) or presence of either FSH or EGF, followed by staining with hematoxylin/eosin. Primary spermatocytes are surrounded by black dashes.

To examine whether EGF is able to stimulate the proliferation of spermatogonia dose-dependently in an endocrine manner in the testis, the testicular fragments containing only spermatogonial stage were cultured for 1 week in the absence or presence of FSH or EGF and subjected to BrdU incorporation assay. As shown in Fig. 1A and 1B, EGF increased the incorporation of BrdU into spermatogonia in a dose-dependent manner, with significant stimulation achieved at the doses of 1000 and 4000 ng/ml. The effect was compatible with that of FSH at 200 ng/ml. Thus, EGF stimulated spermatogonial proliferation in the testis. On the other hand, unlike FSH, EGF could not induce proliferation of Sertoli cells in the spermatogonial stages (data not shown).

To test whether EGF is able to stimulate the differentiation of spermatogonia into primary spermatocytes in the testis, the organs containing spermatogonial stage were cultured for 2 weeks in the absence or presence of FSH or EGF and observed for appearance of primary spermatocytes. As shown in Fig. 1C, in contrast to FSH (200 ng/ml), EGF could not stimulate the appearance of spermatocytes at 4000 ng/ml. Rather, dead cells were detected in the area of the testis containing the 7th generation of spermatogonia treated with EGF as well as the control, but not with FSH. Thus, EGF seemed not to stimulate spermatogonial differentiation into primary spermatocytes in the testis.

### Cloning of EGF receptors in the testis

**Figure 2:**
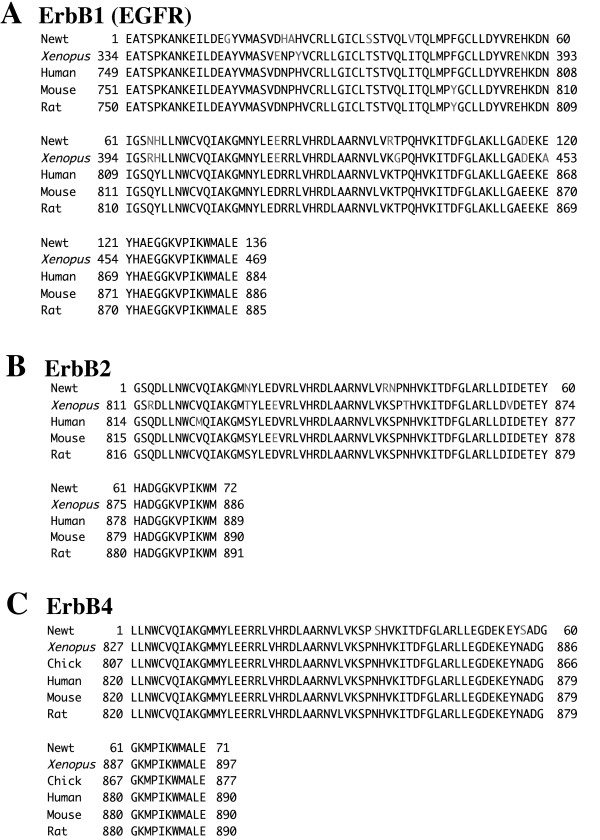
Cloning of the receptors for EGF. The deduced amino acid sequences of ErbB1 (A), ErbB2 (B), and ErbB4 (C), whose partial cDNA clones were isolated from newt testis containing spermatogonial stage by RT-PCR, were aligned with those from mammals and *Xenopus laevis*. The residue numbers are shown at the both sides. The positions where the amino acid residues of ErbB1, ErbB2, and ErbB4 are identical and distinct among species are indicated in black and shaded letters, respectively.

To clarify how EGF stimulated proliferation of the spermatogonia, we first cloned the cDNA for EGF receptors from the testis by RT-PCR using total RNA prepared from testes containing spermatogonial stage. ErbB1 (also known as EGFR) comprises homodimer by itself and heterodimer with ErbB2, ErbB3, and ErbB4 to mediate the signals [41]. Therefore, PCR cloning was performed for these receptors and EGF from newt testis on the basis of the homology to the nucleotide sequences of those that have been previously isolated from other species. The partial cDNA clones containing the highly conserved tyrosine kinase domain of ErbB1, ErbB2, and ErbB4 were isolated, and their predicted amino acid sequences exhibited approximately 92%, 90~96, and 97% identity with those of *Xenopus laevis *and mammals, respectively (Fig. 2A, B, and 2C). In this study, however, we have been unable to clone ErbB3 and EGF cDNA from the testis.

### Expression of the EGF receptors in the testis

**Figure 3:**
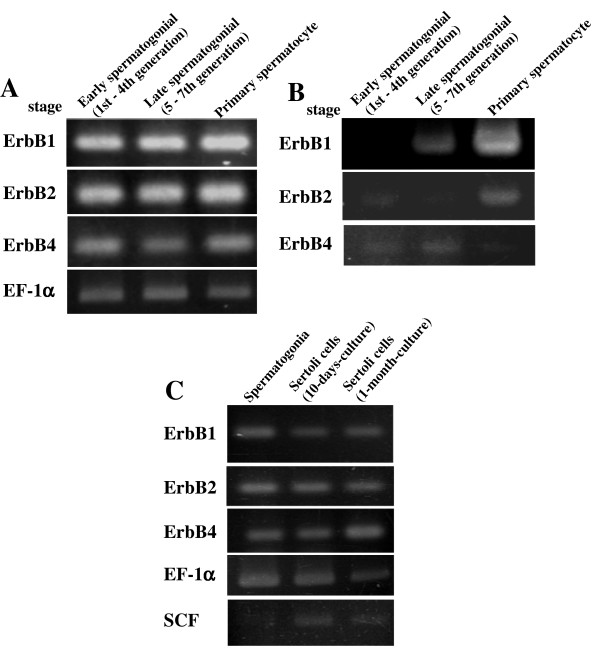
Expressions of mRNA for ErB1, ErbB2, and ErbB4 in various spermatogenic stages and testicular cell types. The expressions of ErbB1 (200 bp), ErbB2 (150 bp), and ErbB4 (150 bp) were analyzed by semi-quantitative RT-PCR. Total RNA was extracted from the testes containing early spermatogonial (1st – 4th generation), late spermatogonial (5th – 7th generation), and primary spermatocyte stages (A and B), and from the fractionated spermatogonia and Sertoli cells, the latter of which were cultured for 10 days and 1 month (C). The cycle numbers in PCR were 35 (A) and 33 (B), and 35 for ErbB1 and ErbB2 and 40 for ErbB4 (C). Elongation factor-1α (EF-1α, 550 bp, 25 cycles) is the internal control. SCF is a Sertoli cell-specific marker. Data are representative of at least 3 independent experiments.

To investigate the relevance of temporal and spatial expressions of the EGF receptors identified from the testis with spermatogonial proliferation induced by EGF, we examined the expressions of ErbB1, ErbB2, and ErbB4 in the spermatogenic stages and cell types by RT-PCR. Total RNA extracted from testes containing early spermatogonial (1st – 4th generation), late spermatogonial (5 – 7th generation), and primary spermatocyte stages was reverse transcribed with random hexamers, and then analyzed by PCR using primers specific for the respective ErbB. The transcripts for all the receptors were present in all the stages examined (Fig. 3A). Both ErbB1 and ErbB2 transcripts appeared less in the spermatogonial stages than in the spermatocyte stage (Fig. 3B, upper and middle panels). On the other hand, ErbB4 mRNA appeared more abundant in the spermatogonial stages than in the spermatocyte stage (Fig. 3B, lower panel).

Testes containing spermatogonial stage were dissociated by collagenase and fractionated into spermatogonia and Sertoli cells by differential adhesiveness to gelatin. Sertoli cell fractions were cultured for long periods (e.g. 10 days and 1 month) to reduce the spermatogonial contaminations. Total RNA prepared from the cell fractions was reverse transcribed and analyzed by PCR as described above. The purity of Sertoli cells in the fraction was evaluated by detecting the mRNA expression of SCF specific for the cells (Fig. 3C, 5th panel). As shown in Fig. 3C, the transcripts for all the EGF receptors cloned were expressed in both spermatogonia and Sertoli cells.

### Functional EGF receptors executed in EGF-induced proliferation

**Figure 4:**
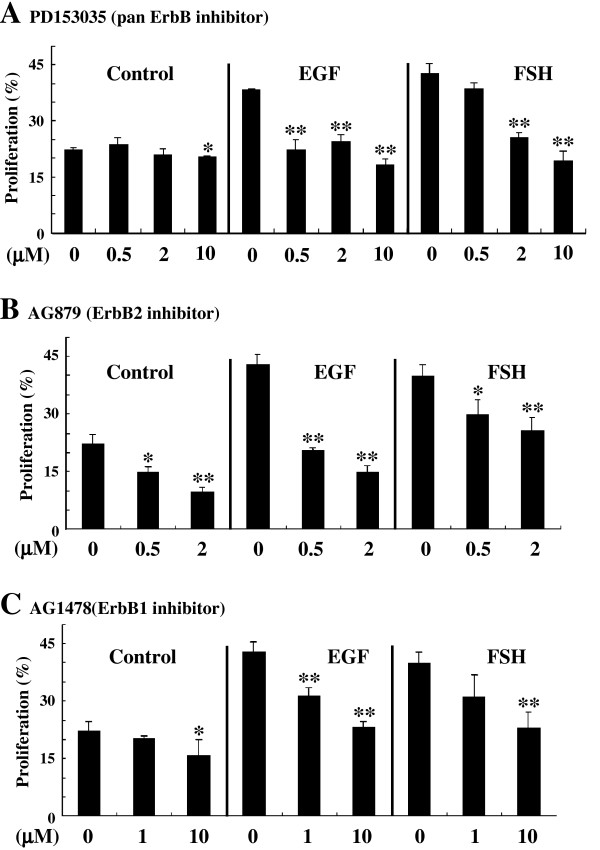
Dose-dependent effects of inhibitors for ErbB on EGF-stimulated spermatogonial proliferation. Testicular fragments containing spermatogonial stage treated without (0) or with various doses of pan ErbB4 inhibitor (PD153035) (A), an ErbB2-specific inhibitor (AG879) (B), or an ErbB1-specific inhibitor (AG1478) (C) were cultured for 1 week in the absence (Control) or presence of either FSH (200 ng/μl) or EGF (4000 ng/μl), followed by BrdU incorporation assay. *, P < 0.05; **, P < 0.01.

Next, to determine which combinations of EGF receptors participate in EGF-induced spermatogonial proliferation, we examined the dose-dependent influences of their inhibitors by using them in the organ culture of the testis treated with EGF. Testes containing spermatogonial stage were cultured for 1 week in the presence of either EGF at 4000 ng/ml or FSH at 200 ng/ml without or together with each of ErbB inhibitors and then subjected to BrdU incorporation assay. As shown in Fig. 4A, EGF-dependent spermatogonial proliferation was suppressed by a pan ErbB inhibitor (PD153035), which inhibits ErbB1 and ErbB4 particularly, at 0.5, 2, or 10 μM remarkably even at the lowest dose (0.5 μM) and completely at the highest dose (10 μM), compared with the control (0 μM). By contrast, FSH-dependent proliferation was dose-dependently suppressed by PD153035 with complete inhibition at 10 μM. A specific ErbB2 inhibitor (AG879) inhibited EGF-dependent proliferation more strongly than FSH-dependent proliferation at the both doses of 0.5 and 2 μM (Fig. 4B). For a specific ErbB1 inhibitor (AG1478), its inhibitory effects on EGF- and FSH-dependent proliferation were almost the same at the both doses of 1 and 10 μM (Fig. 4C).

### Functional intracellular signaling pathways executed in EGF-induced proliferation

**Figure 5:**
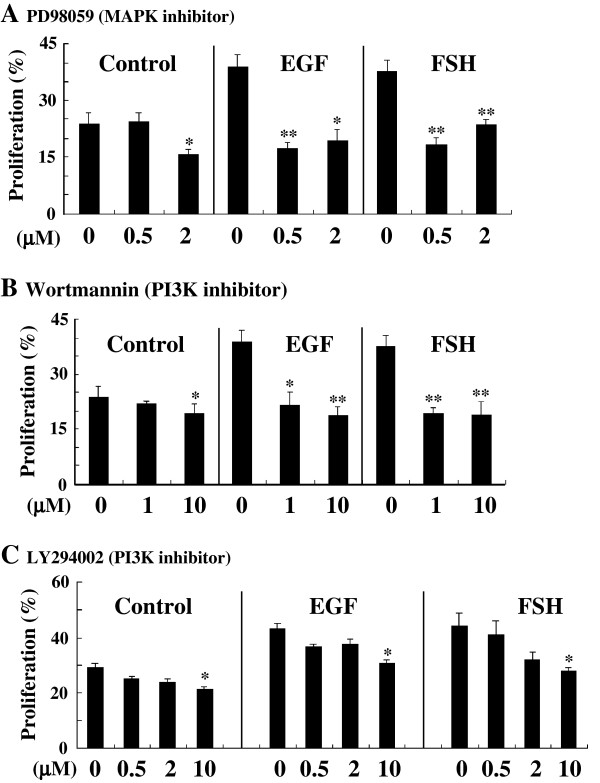
Dose-dependent effects of inhibitors for MAPK and PI3K on EGF-stimulated spermatogonial proliferation. Testicular fragments containing spermatogonial stage treated without (0) or with various doses of a MAPK-specific inhibitor (PD98059) (A) or PI3K-specific inhibitors (Wortmannin (B) and LY294002 (C)), were cultured for 1 week in the absence (Control) or presence of either FSH (200 ng/μl) or EGF (4000 ng/μl), followed by BrdU incorporation assay. *, P < 0.05; **, P < 0.01.

Furthermore, to identify intracellular signal transduction pathways implicated in EGF-induced spermatogonial proliferation, we tested the dose-dependent influences of the inhibitors for MAPK and PI3K in the organ culture of the testis treated with EGF. It is reported that MAPK and PI3K cascades function downstream of EGF signaling [42,43]. Testes containing spermatogonial stage were cultured for 1 week in the presence of either EGF at 4000 ng/ml or FSH at 200 ng/ml without or together with a MAPK inhibitor (PD98059) and PI3K inhibitors (Wortmannin and LY294002) at 0.5, 2, or 10 μM and then subjected to BrdU incorporation assay. As shown in Fig. 5A and 5B, EGF- as well as FSH-dependent spermatogonial proliferation were lowered by a half in the presence of PD98059 and Wortmannin relative to the control (0 μM). LY294002 barely suppressed either of EGF- and FSH-dependent proliferation (Fig. 5C).

### Effect of EGF on the expressions of mRNA for neuregulin1, ErbB family members, SCF, and c-kit

**Figure 6:**
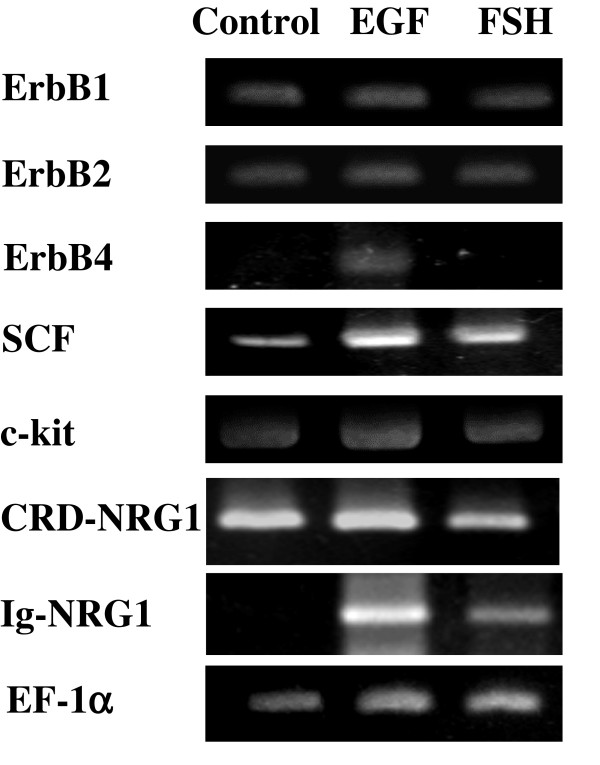
Effects of EGF on mRNA expressions for the EGF receptors, SCF, c-kit and neuregulin1. Total RNA was extracted from the testicular fragments containing spermatogonial stage cultured for 1 week in the absence (Control) or presence of either EGF (4000 ng/μl) or FSH (200 ng/μl), and analyzed by RT-PCR for expressions of ErbB1 (200 bp, 35 cycles), ErbB2 (150 bp, 35 cycles), ErbB4 (150 bp, 35 cycles), SCF (390 bp, 33 cycles), c-kit (200 bp, 40 cycles), CRD-NRG1 (300 bp, 45 cycles), and Ig-NRG1 (450 bp, 45 cycles). EF-1α (550 bp, 25 cycles) is the internal control.

To explore the molecular mechanism by which spermatogonial proliferation was stimulated by EGF signaling, we analyzed changes in the expression of the EGF receptors, their ligands Immunoglobulin-like domain containing neuregulin1 (Ig-NRG1) and cysteine-rich domain containing neuregulin1 (CRD-NRG1), c-kit, and its ligand SCF in the organ cultures of the testes treated with EGF by RT-PCR. It has been reported in mammalian [11,12] and newt testis (Abe et al., in preparation) that SCF is upregulated in Sertoli cells by FSH and enhances spermatogonial proliferation via c-kit. Both Ig-NRG1 and CRD-NRG1 belong to EGF-like peptide family. Recently, Ig-NRG1 has been shown to be upregulated in Sertoli cells in response to FSH and stimulate spermatogonial proliferation in newt tesits (Oral et al., submitted). Testes containing spermatogonial stage were cultured for 1 week in the absence or presence of EGF or FSH and used for total RNA extraction. Interestingly, RT-PCR analyses showed that the expression of ErbB4 mRNA was clearly promoted by only EGF (Fig. 6, 3rd panel), and that of SCF mRNA was significantly promoted by not only FSH but also EGF (Fig. 6, 4th panel). In addition, Ig-NRG1, but not CRD-NRG1, was more drastically upregulated by EGF than by FSH (Fig. 6, 7th panel). The expressions of other genes examined remained unchanged in the testes treated with FSH and EGF compared to the control.

## Discussion

In this study, EGF has been shown to stimulate spermatogonial proliferation, but not their differentiation into spermatocytes, in newt testis harboring germ cells enclosed in cysts with the blood-testis barrier consisting of Sertoli cells. This result was consistent with that in mammalian spermatogonia [27,34,35], suggesting that the stimulatory effect of EGF on spermatogonial proliferation was highly conserved beyond species. However, unlike mouse spermatogonia, which are located outside of the blood-testis barrier, newt spermatogonia are enclosed by Sertoli cells and located inside of the blood-testis barrier, so that EGF could not act on spermatogonia directly like an endocrine manner. In addition, EGF seemed not to stimulate Sertoli cell proliferation (data not shown), regardless of requirement for Sertoli cells in EGF-induced spermatogonial proliferation. These findings suggested that EGF could function as an indirect trigger specific for spermatogonial proliferation by altering gene expression for some direct stimulators locally within the cysts. Therefore, EGF may be defined as an additional endocrine factor, which could regulate spermatogonial proliferation in concert with endocrine factors like FSH [13-15], in newt testis.

Conversely, when the organ culture of mammalian testes, in which spermatogonia have a potential to be the direct target for endocrine factors, is exposed to added factors, it is unclear whether the factors stimulate spermatogonial proliferation directly in a paracrine manner or indirectly via somatic cells including Sertoli cells in an endocrine manner [23,33-35]. Thus, the mechanism of action of hormones and growth factors on spermatogonia remained incompletely understood in the testis of newt as well as of other species. Their endocrine effects could be investigated with newt testis, in which spermatogonia should never be the direct target for endocrine factors. Therefore, clarifying the physiological mechanism of action and function of EGF in such an organism seems linked to elucidating its endocrine function in unamniotes.

The present analyses by RT-PCR show that the EGF receptors, ErbB1, ErbB2, and ErbB4, are localized in Sertoli cells as well as spermatogonia at the spermatogonial and spermatocyte stages examined, indicating that the receptors expressed in Sertoli cells are initially responsive to EGF because it (molecular weight of approximately 6 kDa) very probably can not penetrate into cysts to act on spermatogonia due to the existence of the blood-testis barrier [6]. Thus, the expression patterns for the EGF receptors could be enough to explain the mechanism of action on Sertoli cells in an endocrine manner. Further evidence for the initial site of action on Sertoli cells of EGF was provided by using ErbB inhibitors as described below.

We examined how the stimulatory action of EGF on spermatogonial proliferation is caused using inhibitors for the EGF receptors, and MAPK and PI3K. An inhibitor specific for ErbB1, AG1478, suppressed spermatogonial proliferation induced by EGF. A similar suppressive effect was seen with an inhibitor specific for ErbB2, AG879, and an inhibitor for ErbB1 and ErbB4, PD153035. Considering the general combinations of the EGF receptors, ErbB1 is thought to form homodimer by itself and heterodimer with ErbB2 and ErbB4 to signal EGF in Sertoli cells, resulting in stimulating spermatogonial proliferation.

EGF-stimulated proliferation was suppressed by each of MAPK inhibitor PD98059 and PI3K inhibitors Wortmannin and LY294002. MAPK and PI3K may be expressed ubiquitously in various cells and are well known to play important roles in the intracellular signaling. These results suggested both kinase cascades function downstream of the EGF receptors in a concert or/and independent manner. However, we can not rule out a possibility that these inhibitors used here are probably able to penetrate the blood-testis barrier into cysts to inhibit the activity of EGF receptors, MAPK, and PI3K, which are localized not only in Sertoli cells but also in spermatogonia, because their molecular weights are less than 500 Da [6]. In such a view, we could believe that the inhibitors for the EGF receptors also suppressed spermatogonial proliferation induced by FSH. Since FSH receptor is expressed only in Sertoli cells, FSH can not stimulate the proliferation directly, supporting the existence of some ligands (EGF-like peptides) for the EGF receptors such as Ig-NRG1 mediating the proliferation stimulating action of FSH that are produced and secreted in Sertoli cells and subsequently triggers the spermatogonial proliferation within the cysts (Oral et al., submitted). The dose responses of suppression in the spermatogonial proliferation induced by EGF to all of the inhibitors for the EGF receptors were slightly stronger than that induced by FSH, suggesting that low doses of the inhibitors are enough to suppress EGF-mediated activity of the EGF receptor in Sertoli cells, but their high doses are necessary to FSH-mediated activity of the EGF receptor in spermatogonia. Anyway, since the EGF receptors are expressed at least in both spermatogonia and Sertoli cells, the inhibitors seemed to block the signaling within the both cell types, consequently inhibiting EGF-dependent spermatogonial proliferation.

For the molecular mechanism stimulating spermatogonial proliferation by EGF, we can easily imagine an idea that Sertoli cells express and secrete some factors locally in response to EGF, and they act on germ cells directly in newt testis. Surprisingly, our data demonstrated first that EGF upregulated the transcripts of SCF, Ig-NRG1, and ErbB4. It is known that SCF is a growth factor for spermatogonia and expressed in Sertoli cells, and its expression is promoted by FSH in mammalian [12] and newt testis (Abe et al., in preparation). Recently, we have shown that Ig-NRG1 expressed in Sertoli cells is upregulated by FSH and mediates FSH-stimulated spermatogonial proliferation within the cysts (Oral et al., submitted). Thus, EGF was shown to mimic, in part, FSH effects on enhanced SCF and Ig-NRG1 expressions and stimulated spermatogonial proliferation potentially, but not on stimulated proliferation of Sertoli cells and other somatic cells, though this biological significance is not understood at all. Therefore, SCF and Ig-NRG1 whose expressions are enhanced in Sertoli cells in response to EGF may be secreted from Sertoli cells locally and then act on germ cells directly to stimulate spermatogonial proliferation in newt testis. Furthermore, EGF increased the ErbB4 expression, but in which testicular cell type this event occurs remains unknown. If an increase in ErbB4 expression occurs in spermatogonia, their responsibility to the ligands (EGF-like peptides) for ErbB4 that are secreted from testicular cells including Sertoli cells might be enhanced, leading to activating the signaling pathways downstream of ErbB4 and then stimulating spermatogonial proliferation more efficiently. On the other hand, if an increase in ErbB4 expression occurs in Sertoli cells and other somatic cells in newt testis, like mammalian testis where the EGF receptors are also found in Leydig, Sertoli, and peritubular cells [35], they might be highly responsive to the ligands for the EGF receptors and secrete different kinds of such ligands, leading to stimulating spermatogonial proliferation consequently. Now we are under investigation about in which cell types the expression of ErbB4 is promoted after EGF stimulation.

## Conclusion

The effect of EGF on spermatogonial proliferation was investigated in the organ culture of newt testis, indicating that it is potentially responsive to EGF in an endocrine manner during spermatogenesis. However, in the testis, endocrine factors are unable to act on germ cells but on somatic cells directly by the existence of the blood-testis barrier [6]. Therefore, we aimed to clarify the mechanism of action of EGF on spermatogonial proliferation. The present study identified the functional receptors, ErbB1, ErbB2, and ErbB4, and intracellular signaling pathways, MAPK cascade and PI3K cascade, in EGF-mediated spermatogonial proliferation. Subsequently, we aimed to explore the mechanism causing the proliferation. This study presents first data that expressions of SCF and Ig-NRG1 are enhanced by EGF as well as by FSH, and that of ErbB4 is enhanced by EGF but not by FSH, in the testis. These data suggest that the EGF-stimulated spermatogonial proliferation is in part mediated by elevating the expression of SCF and Ig-NRG1 via the EGF receptors in Sertoli cells.

## Authors' contributions

KA carried out all the experiments. KE participated in the design and coordination of the study, and drafted the manuscript. SA conceived of the study, participated in its design, and completed the manuscript.
